# Detecting and Quantifying Variability in Mass Spectrometry-Based Metabolomics Datasets

**DOI:** 10.3390/biom16101399

**Published:** 2026-09-25

**Authors:** Aleesa E. Chua, Heather Desaire

**Affiliations:** Department of Chemistry, University of Kansas, Lawrence, KS 66045, USA; hdesaire@ku.edu

**Keywords:** mass spectrometry, variation, quality control, visualization, statistics, data analysis, metabolomics

## Abstract

Mass spectrometry-based metabolomics approaches generate high-dimensional datasets that enable comprehensive profiling of metabolites and other biomolecules. However, these datasets are influenced by multiple sources of variability, including both the biological variation of interest and unwanted technical or systematic variation introduced throughout experimental or analytical workflows. Distinguishing between these sources of variation is important for ensuring robust and reliable downstream interpretation. This review examines commonly used approaches for the detection and quantification of variability in MS-based omics datasets, including statistical and multivariate methods such as coefficient of variation analysis, principal component analysis, variance-based metrics and other quality assessment and visualization techniques used to evaluate data structure and reproducibility. The review further highlights how these approaches can be applied to identify systematic sources of variation to support the appropriate implementation of normalization strategies. Improved detection and characterization of variability enhance data quality, reproducibility, and interpretability, which thereby support more reliable biomarker discovery and broader omics applications.

## 1. Introduction

Mass spectrometry (MS) has enabled the comprehensive detection of a wide range of metabolites in complex biological samples with high sensitivity and high throughput. MS analyses offer a way to identify potential analytes that are discriminative of disease states, treatment responses, and physiological conditions [1,2]. In this context, MS-based omics studies have contributed significantly to advancing our understanding of disease progression and the underlying biological alterations associated with it [3,4].

Just as disease onset and progression arise from many contributing factors, MS-based omics data are similarly influenced by multiple sources of variation. In this review, variability broadly refers to differences in measured metabolite responses across samples or replicate measurements, which may be described quantitatively using measures such as the coefficient of variation. Depending on the analytical workflow, these responses may include ion intensities, peak areas, normalized abundances, or metabolite concentrations determined using internal standards and calibration. As shown in Figure 1, these sources of variation may be biological, technical, analytical, or systematic in nature and can be introduced at each stage of the experimental workflow [5,6,7,8,9]. Consequently, each measured metabolite response may reflect not only the underlying variation of interest but also a combination of technical and experimental effects. Furthermore, the diversity of these sources of variation, as well as the range of patterns they may exhibit, from simple linear effects to more complex nonlinear structure, makes comprehensive characterization of variability challenging [10,11]. A single strategy is therefore unlikely to be sufficient for capturing all relevant forms of variability within an MS-based omics dataset. Yet, the characterization of variability remains important, as failure to identify and account for unwanted variability can obscure true biological differences and compromise statistical interpretation [12,13]. Detecting variability is therefore a crucial step as it informs whether additional data processing, normalization, batch correction, or even redesign of the experimental workflow is needed to mitigate unwanted effects.

Ultimately, variability in metabolomics experiments is inherent and unavoidable, highlighting the need to understand both the sources and magnitude of variability present within MS-based datasets. Previous reviews have provided extensive guidance on strategies for addressing unwanted variation, including normalization, batch correction, quality control, and other data-processing approaches [10,11,14,15]. In contrast, the present review focuses on the step that precedes these mitigation strategies: determining whether variability is present, identifying its potential source, and quantifying its contribution to the overall variation in a dataset. This information is important for determining whether an observed source of variability represents unwanted technical or experimental variation that should be addressed or meaningful biological variation that should be retained and considered during downstream analysis. Once unwanted variability has been identified and characterized, existing reviews provide guidance on selecting appropriate strategies for mitigating its effects [10,11,14,15]. Together, these approaches can support more informed data processing and interpretation and promote more reliable and reproducible MS-based metabolomics analyses.

## 2. Methods for Detecting Variability

### 2.1. The Case for Detecting Variability

In comparative metabolomics studies, the ability to identify biologically meaningful differences between experimental groups is primarily influenced by the relationship between within-group and between-group variability. Between-group variability reflects differences in metabolite levels between experimental groups, while within-group variability reflects the spread of measurements among samples within the same group. The relative magnitude of these two sources of variability ultimately determines how effectively groups can be distinguished.

Figure 2 illustrates this concept using a simplified example in which the abundance of a single metabolite is measured across healthy and diseased sample groups. In this case, statistical tests, like a *t*-test, can be used to determine whether there is an observable difference in the mean metabolite intensity between the two conditions [16,17]. As shown in Figure 2A, the two groups are most effectively differentiated when between-group variability is high relative to within-group variability. In contrast, increased within-group variability (Figure 2B) or reduced between-group variability (Figure 2C) can substantially limit the discriminative ability. When both between-group variability and within-group variability are small, the absence of clear separation may simply reflect the fact that no meaningful biological difference exists between the healthy and diseased groups for the measured metabolite. However, when both between-group and within-group variability are large (Figure 2D), the elevated within-group variability can obscure true differences between the two sample groups, potentially leading to inaccurate interpretations of the data.

This problem is further exacerbated in more complex experiments, like untargeted MS-based omics analyses, wherein hundreds to thousands of metabolites are measured simultaneously, each of which is potentially influenced by various sources of variability. For example, while the biological condition under investigation (healthy vs. diseased) contributes to between-group variability, biases in the experimental design, such as the lack of randomization, non-stratified sample selection, or batch effects, may introduce systematic differences that artificially inflate observed between-group variation [18,19,20]. Similarly, variability introduced during sample preparation, processing, MS analysis, and even data processing can increase the observed within-group variation [6,21,22,23].

The complexity of these MS data sets can thus make it difficult to ensure that all sources of variability are adequately controlled for. Consequently, detecting and characterizing variability in MS-based omics datasets is important. Without this understanding, observed differences between groups may reflect extraneous sources of variation rather than the true biological effect of interest.

### 2.2. Common Methods for Detecting Variability

Given the impact that unwanted variability can have on the interpretation of omics data, evaluating sources of variability has become a routine component of MS-based omics workflows. Among the most used approaches for detecting variability are principal component analysis and statistical metrics such as the coefficient of variation.

#### 2.2.1. Principal Component Analysis

Principal Component Analysis (PCA) is one of the most common tools for exploratory data analysis. Its heavy usage can largely be attributed to its ability to present complex, high-dimensional datasets in an intuitive way by reducing them to a smaller set of variables, termed ‘principal components,’ that capture the major sources of variation in the data [24,25].

These principal components (PCs) are formed via linear combinations of the original features, where the contribution of each feature to a given component is represented by a weight, referred to as a loading. The first principal component (PC1) captures the direction of greatest variance in the dataset, while the second principal component (PC2) captures the next largest source of variance, and so on. A high-level visualization of the data can then be obtained by projecting the data onto the first two or three PCs in a score plot, where each sample’s mass spectrum is represented as a single data point and the resulting clustering patterns reveal potential sources of variability. Importantly, PCA is an unsupervised technique, meaning that in any case, clustering patterns are determined solely by the dominant sources of variation within the dataset rather than being influenced by predefined sample labels [26]. When clustering patterns are observed, the corresponding feature loadings can provide further insight into which variables are most influential in driving that variability [27].

PCA is commonly incorporated in quality control (QC) applications, where analyses are performed on datasets that include both study samples and QC samples [28,29,30]. The resulting score plots are evaluated to assess the quality of the corresponding MS data. In this context, tight clustering of QC samples relative to the biological samples in the PCA space is desirable (Figure 3A), as it indicates high analytical reproducibility and stable instrument performance [30,31,32]. Conversely, if QC samples are spread across the PCA plot, this may suggest that technical variability, in the form of batch effects, instrumental drift or other systematic errors, is contributing substantially to the overall variation.

PCA can similarly be applied to datasets containing only study samples. When the biological variable of interest represents the largest source of variation, samples may cluster according to experimental groups (Figure 3B). PCA has been used to distinguish samples of different treatment groups, disease states, and other biological classifications [31,33,34,35]. However, if the between-group variation associated with this variable is smaller, or if other sources of variability are more pronounced, alternative clustering patterns may emerge (Figure 3C). Clustering by acquisition order, analytical batch, or sample preparation group, for example, may indicate the presence of unwanted systematic bias [13,36]. So long as sufficient metadata is collected, samples can be colored according to different variables on the score plot to help reveal which factors contribute most strongly to the first few principal components. In this way, PCA provides a convenient way of identifying dominant sources of variability within MS-based omics datasets.

In addition to visual interpretation of the score plot, several quantitative measures can provide further information about the observed variability. The proportion of variance explained by each principal component indicates how much of the overall variability in the dataset is represented by that component. Sample scores provide the coordinates of individual samples along each principal component and can be used to calculate quantitative measures of sample similarity or separation. Loadings describe the contribution of individual features to each principal component and can therefore help identify which features contribute most strongly to the observed patterns of variability. These measures provide additional quantitative information that can support the interpretation of clustering and separation observed in PCA score plots.

#### 2.2.2. Coefficient of Variation/Relative Standard Deviation

PCA-based analyses are often complemented by quantitative metrics used to assess reproducibility [30,37]. One such measure is the coefficient of variation (CV), also referred to as the relative standard deviation (RSD), which quantifies the variability of metabolite feature intensities relative to their mean. It is calculated as the standard deviation divided by the mean intensity, and when expressed as a percentage, it is expressed as %RSD. CV is mostly used to evaluate measurement precision and to characterize analytical variability. Lower CV or RSD values generally suggest more reproducible measurements, while higher values reflect increased variability [30,31,38]. In many metabolomics studies, thresholds of approximately 15-30% are used to evaluate analytical reproducibility, with features exceeding these thresholds often considered to be strongly influenced by extraneous sources of systematic variation and therefore less reliable [39,40].

Typically, RSDs are calculated across replicate or quality control (QC) samples. In this context, the type of replicate and the way QC samples are prepared dictate the information conveyed by the resulting CV values. CVs calculated across biological replicates reflect inherent biological variation, while CVs derived from technical replicates or QC samples capture analytical or systematic variation. Similarly, the timing and preparation of QC samples can provide insight into the reproducibility of specific stages of the experimental workflow. For instance, Yu et al. [41] used QC RSDs to compare the reproducibility of different extraction methods and Zhang et al. [42] evaluated peak intensity CV distributions across different sample preparation strategies. Beyond guiding workflow optimization, CVs can also be used to identify the sources of variability. For example, Burgos et al. [43] used QC RSD values to evaluate signal variability in an untargeted LC-MS metabolomics analysis of human urine. Increased variability was associated with the accumulation of sample components in the column and ion source, while modifications to the analytical workflow reduced RSD values for several metabolites from approximately 60% to 10%. CVs can also be examined collectively to provide a broader view of variability across a dataset. Distributions of feature-level CVs have been used to compare variability across different sample matrices and organisms and can be visualized using boxplots, histograms, or density plots [38].

#### 2.2.3. Limitations of These Methods

The value of PCA and CV/RSD is reflected in their inclusion in several software tools for data processing and analysis, where they are widely used as frontline measures of data quality [44,45,46]. Despite this, there remain some important limitations that restrict their ability to provide a comprehensive understanding of variability.

Firstly, while PCA is valued for its interpretability, this same feature also limits the scope of variability that can be detected under this approach. PCA score plots are typically presented as 2D or 3D projections of the data, which intuitively capture the dominant sources of variation. However, interpretations based on only the first few principal components may fail to capture smaller, yet still meaningful, patterns of variability represented by higher-order PCs [47,48]. In the context of exploratory data analysis, focusing on these dominant sources of variation may be sufficient, but for other applications, such as in informing data processing and normalization decisions, a more comprehensive understanding of variability is needed. Furthermore, the unsupervised nature of PCA can make it difficult to determine the origins of the observed variation [49,50]. Although clustering patterns may be readily apparent, their underlying causes are often difficult to interpret without sufficient metadata. In addition, PCA is largely qualitative and interpretation of clustering can be subjective, which may further complicate interpretation [13]. As a result, clustering or separation observed in a PCA score plot should not be used alone to attribute the observed variability to a specific experimental or biological factor. Other factors may contribute to similar clustering patterns, particularly when variables are correlated or confounded. Therefore, PCA should be interpreted alongside relevant experimental metadata and, when appropriate, complementary quantitative approaches to better determine the source of the observed variability.

Secondly, the coefficient of variation provides a limited view of variability, as it primarily quantifies measurement precision rather than capturing the full range of variability present in a dataset. While CVs are effective for identifying systematic technical variation, especially in the form of batch effects or analytical drift, they do not necessarily account for other sources of variability that may affect all samples uniformly [30,37,51]. In addition, CV values can be influenced by multiple confounding factors, making it difficult to attribute this observed variation to a specific source. CV calculations are also biased toward highly abundant and consistently detected features. Low abundance or infrequently detected metabolites often exhibit higher CV values due to lower S/N, whereas high abundance features appear to be more reproducible [52]. However, a low CV does not necessarily guarantee that such features are free from the influence of extraneous sources of variability. Together these limitations demonstrate the need for complementary approaches that can more comprehensively characterize variability in MS-based omics datasets.

### 2.3. Other Methods

#### 2.3.1. Extensions for Classic PCA

To address the qualitative nature of PCA, quantitative distance-based metrics are often employed [53,54,55]. One common approach includes calculating Euclidean distances between samples in the PCA space. The Euclidean distance between two samples is calculated as the square root of the sum of the squared differences between their scores across the principal components included in the analysis. Larger distances indicate greater dissimilarity, while smaller distances indicate greater similarity between samples. For example, Huang et al. [56] calculated Euclidean distances among QC samples across different tissue types and derived average intragroup and intergroup distances. Here, shorter intra-group distances reflect greater similarity within a group, while larger intergroup distances indicate clearer separation between groups. While this approach can provide a quantitative framework for assessing clustering patterns beyond subjective visual interpretation, they remain limited by the lack of standardized thresholds for defining what a ‘meaningful’ separation between groups is. Furthermore, as clustering patterns in PCA can depend on the choice of PC used for visualization, alternative strategies have been proposed. For instance, PC-paired boxplots have been used to provide more consistent comparisons across components. Kellogg et al. demonstrated that group separation and outlier detection can vary depending on which PC are plotted, highlighting the risk of relying on a single PCA score plot alone [57]. Figure 4A,B exemplify this problem, where the apparent separation between groups varies depending on the combination of PCs visualized. PC-paired boxplots (Figure 4C) provide a complementary representation by displaying group distributions across individual PCs, making it easier to identify which components contribute to the observed separation. This approach has also been used to distinguish different sources of variability, such as batch and class effects, that may be captured by different PCs [58]. While PC-paired boxplots do not eliminate the qualitative nature of PCA interpretation, they can help identify which PCs contribute most strongly to the separation between groups.

#### 2.3.2. Hierarchical Clustering Analysis Dendrograms

Hierarchical Clustering Analysis (HCA), like PCA, is an unsupervised technique used to group similar observations within a dataset [59,60]. These groupings can be generated using either a bottom-up (agglomerative) or top-down (divisive) approach. In agglomerative clustering, samples start as individual clusters and are progressively merged into groups based on how similar they are, while in divisive clustering, all samples start in a single cluster and are successively divided into smaller groups [61]. HCA groups samples by comparing their overall feature patterns, so that more similar samples cluster together. These cluster groupings are graphically depicted by a dendrogram, which shows how closely related samples are and how they group together at different levels of similarity. The dendrogram’s tree-like structure makes groupings easier to interpret, and because it can be examined at multiple levels, HCA can reveal both broad clustering patterns [57,58,59,60,61,62] and more subtle subgroup structure, thereby facilitating the identification of sources of variability that may operate at different scales within the dataset [63]. An example dendrogram generated from HCA of simulated data is shown in Figure 5, where the largest branches separate the two biological groups, followed by smaller divisions associated with processing batch and tighter clustering among replicate samples. Additionally, both the presence of cluster groupings and the lack of expected clustering can be informative. For example, HCA dendrograms have revealed clustering associated with processing batch and instrument-related shifts whereas the failure of replicate injections to cluster has pointed to the presence of chemical interferents [64,65]. In both cases, these patterns guided targeted mitigation strategies aimed at improving data quality.

#### 2.3.3. Distribution and Trend Plots

Distribution and trend plots can also be used to detect sources of variability in MS-based omics datasets. Different plot types can reveal different forms of variation across samples, groups, or run order. For example, plotting QC signal against the injection order can reveal temporal effects, like instrumental drift (Figure 6A) [30,66]. Similarly, boxplots, violin plots, density plots, or histograms of sample intensities can be used to identify global distributional differences or other systematic effects across batches or sample groups (Figure 6B) [67,68]. Relative log abundance (RLA) plots can also be used to visualize systematic variation in metabolite intensities (Figure 6C) [69]. Two types of RLA plots can be made: within-group RLA, which assesses the consistency of samples within the same sample group, and across-group RLA, which compares metabolite measurements across distinct sample groups. In both cases, departures from the expected centered distribution can indicate the presence of systematic effects. These visualization-based approaches provide a simple and interpretable way of identifying potential sources of variability. However, they are primarily descriptive, do not directly quantify the contribution of specific sources of variation, and often depend on prior knowledge of which factors may be relevant to examine.

#### 2.3.4. D-Ratio

Calculating the dispersion ratio (D-Ratio) is one way to consider both the technical variation captured by QC samples and the biological variation among study samples [30,51,70]. For a given metabolite, i, the D-Ratio can be calculated as:

$${Dispersion\;Ratio}_{i}=\frac{\sqrt{\sigma_{i,QC}^{2}}}{\sqrt{\sigma_{i,sample}^{2}+\sigma_{i,QC}^{2}}}\times 100\%,$$

where $\sigma_{i,QC}^{2}$ denotes the variance of replicate QC samples (approximating the technical variability) and $\sigma_{i,sample}^{2}$ denotes the variance of the study samples (reflecting the biological variability). Thus, D-Ratio values provide a measure of the relative contribution of QC dispersion compared with biological dispersion, with values near 0% indicating the sole contribution of biological variability and values near 100% reflecting a stronger influence of technical or unwanted variability.

The key strength of the D-Ratio is that it shows whether technical or biological variability contributes more to the spread of the data. By calculating the D-ratio across individual features, specific metabolites can be identified where unwanted variation exceeds biological variation, which could serve as a basis for filtering unstable features [71]. Calculating the D-ratio across the whole dataset, on the other hand, can be used to summarize the overall quality of the dataset. A major limitation, however, is the lack of a universally established threshold for what makes an acceptable D-ratio. This can make its interpretation difficult, and as such, the D-ratio is often applied as a complement to other metrics, such as the CV.

#### 2.3.5. Intraclass Coefficient (ICC)

Correlation-based metrics are also frequently used to evaluate reproducibility [52,72]. Pearson correlations, for example, can be calculated between replicate measurements or QC samples to evaluate the consistency of measurements across different analyses. The Pearson correlation measures how similarly two sets of measurements vary, producing a correlation coefficient, r. The coefficient is calculated from the covariance between the two sets of measurements, scaled by the variability within each set. Correlation coefficients range from −1 to 1, with values approaching −1 or 1 indicating stronger negative or positive linear relationships, respectively, and values near 0 indicating little to no linear relationship. Recently, however, the intraclass correlation coefficient (ICC) has been proposed as a more discriminatory alternative, as it can detect effects such as drift and scaling that Pearson correlation may not capture [52].

The ICC is based on decomposition of the total variance of each metabolite into between-sample variance, within-sample variance, and technical variance, with the latter representing variability introduced by laboratory procedures. In this framework, technical ICC is defined as the proportion of total variance attributable to biological variation, calculated as the sum of between- and within-sample variance divided by the total variance. High ICC values therefore indicate that technical variance is low relative to biological variance, suggesting good analytical precision and reproducibility. Generally, ICC values less than 0.4 indicate poor reliability, while ICC values exceeding 0.75 are indicative of excellent reliability [73,74,75]. ICC is commonly applied to longitudinal or epidemiological studies to assess the reliability of metabolite measurements over time by comparing variability between individuals with variability within individuals [74,76,77]. In MS-based studies, ICC has been used to evaluate overall laboratory reproducibility through calculation of the median technical ICC across all detected features in serum lipidomic datasets [7]. In addition, the resulting estimates of variability have been used to inform statistical power calculations for given effect sizes, thereby guiding the design of future lipidomic studies. ICC has also been applied to evaluate the effects of preanalytical variables on sample stability [23].

## 3. Methods for Quantifying Variability

### 3.1. The Case for Quantifying Variability Contributions

While the methods described above enable the detection of variability, they provide limited insight into each source of variability’s relative magnitude and contribution to overall variation. Additionally, a major limitation of these approaches is their subjectivity. For example, while PCA clustering patterns or high CVs may suggest the presence of systematic variability, they do not clearly establish whether data normalization and correction are required. This uncertainty complicates decisions about effective data processing, since the wrong approach may either fail to effectively remove unwanted variability or inadvertently remove too much of the meaningful signal [78].

To address this limitation, several statistical approaches were developed to facilitate a clearer understanding of the overall variability within a dataset. Many of these methods were originally designed to probe the significance of biological variation in cases where it was masked by other, more dominant sources of variation in exploratory data analysis tools, such as PCA. However, as these approaches enable the quantification of the individual contributions of many explanatory variables, whether biological or technical, such tools can be similarly applied to the detection of unwanted variability. Under this application, these quantitative measures can provide a more objective way to decide if correction is necessary, the appropriate strategy to use, and even how experimental design might be improved to minimize unwanted variability in future studies.

Despite these advantages, the quantification of variability is still relatively underutilized in metabolomics, due to the greater simplicity of the traditional variability detection methods. Additionally, more effort is needed for quantification; detailed metadata is often required, analyses can be computationally demanding, and it can require a higher level of statistical expertise. Still, these quantitative approaches are important as they can provide valuable insight into the structure of variability within a dataset and can reveal which stages of the workflow contribute most strongly to the overall variability.

### 3.2. Methods for Quantifying Variability

#### 3.2.1. Guided PCA and the δ-Statistic

Guided PCA (gPCA) was initially developed by Reese et al. in 2013 to quantify the existence of batch effects in genomic data [79]. Unlike traditional (unguided) PCA, which identifies directions of greatest variation solely from the raw data, gPCA incorporates external information about sample batches. The analysis is first “guided” by an indicator matrix that specifies which batch each sample belongs to. This indicator matrix is transposed and used to combine the data so that the information is summarized at the batch level rather than the individual level. PCA is then applied to this batch-based version of the data, allowing the main sources of variation to be interpreted in terms of differences between batches rather than only among individual samples. To quantify the impact of batch effects, Reese et al. introduced a test statistic, δ [79]. This statistic is defined as the ratio between the variance explained by the first principal component in gPCA and that explained by the first principal component in standard PCA. A higher δ indicates stronger batch effects, and its statistical significance can be evaluated through permutation testing. Determination of a significant effect can therefore motivate the application of batch-correction methods during data processing to reduce the influence of unwanted systematic variation.

Beyond genomics, this approach has been utilized in both proteomic and metabolomic applications in tandem with other approaches (such as traditional PCA and internal standard drift detection) to determine within-group and between-group batch effects [49,80,81,82]. Although gPCA has been used predominantly for batch effect detection, it is not limited to MS instrumental drift detection. By redefining the indicator matrix with other sample-level metadata, gPCA can be used to detect other structured sources of variability [79]. For instance, in one proteomics study, gPCA was used to evaluate the influence of sample collection day and tube type [82].

#### 3.2.2. Principal Variance Component Analysis

Principal Variance Component Analysis (PVCA) addresses a limitation of traditional PCA, wherein detection of unwanted variability is complicated by qualitative analysis and the increasing difficulty of interpretation with numerous potential sources of variability. PVCA combines PCA with variance component analysis (VCA) to estimate how much individual biological or technical factors contribute to the overall variability in a dataset [83,84]. In this approach, PCA is first applied, and a subset of principal components explaining a substantial proportion of the total variance in the dataset is retained for further analysis. Then, linear mixed models are fitted to these retained principal components, with explanatory variables corresponding to potential sources of variability included as random effects. The contribution of each explanatory variable is estimated for each principal component, and these estimates are weighted by the proportion of total variance explained by that component. The weighted contributions are then combined across components to obtain an overall estimate of the variance attributable to each explanatory variable.

The biggest advantage of PVCA is that it provides a clear, quantitative metric that estimates the relative magnitude of each source of variability. These estimates can be directly visualized as standardized variance components in pie charts or bar plots, facilitating straightforward comparison across explanatory variables or factors (Figure 7). An additional benefit is that PVCA not only quantifies the contributions of individual sources but also their interactions, making it possible to visualize complex variance structures in an intuitive way.

This technique has frequently been applied to quantify the presence of batch effects, but its utility extends to broader characterizations of experimental variability [63,85,86,87]. For example, in one LC-MS metabolomics study of children sampled before and during Plasmodium falciparum infection, PVCA was used to evaluate the relative contributions of several biological and experimental factors to the observed metabolomic variation, including infection status, age, sex, metabolomics run day, hemoglobin level, lymphocyte-to-neutrophil ratio, and individual effects [88]. In this study, PVCA complemented PCA by not only revealing dominant patterns of variation in the dataset but also quantifying the relative contributions of the factors underlying that variation. The insights gained from PVCA have also been used to compare analytical platforms to determine whether observed variance is driven primarily by biologically meaningful factors, such as patient effects, or by technical factors introduced by the platform [89]. In this way, it can help identify the platform with the greater ability to capture biologically meaningful differences among clinical samples.

#### 3.2.3. Principal Component Partial R-Square

Principal Component Partial R-square (PC-PR2) is a method that stems from PVCA that similarly detects and quantifies the contributions of different sources of variability to the overall variation in a dataset [90]. As in PVCA, the analysis begins with PCA for dimensionality reduction, and the first k principal components explaining a predefined proportion of the total variance, typically 80%, are retained. The projection scores for these retained principal components are then used as dependent variables in multivariable linear regression models, with the explanatory variables, or covariates, included as independent variables. The main difference between PC-PR2 and PVCA lies in this modeling step, where PC-PR2 uses multivariable linear regression and PVCA uses mixed models with random effects. This difference allows PC-PR2 to accommodate intercorrelation among covariates more readily, among other advantages noted in the original Fages paper [90]. An R-square statistic can then be calculated from each regression model. Specifically, R_partial_^2^ values are calculated to quantify the unique contribution of each explanatory variable to a given principal component, conditional on all other covariates in the model. These R_partial_^2^ values are then weighted by the proportion of variance explained by each retained component and averaged across components, yielding a single statistic that reflects how strongly each explanatory variable contributes to the overall variation in a dataset. Higher PC-PR2 values thus indicate stronger contributions.

PC-PR2 shares many of the same advantages of PVCA, particularly in providing an easily interpretable quantitative summary of the contribution of different sources of variability. The weighted R_partial_^2^ can be easily visualized as bar plots, facilitating straightforward identification of the largest contributors to variation (Figure 7). In addition, significance testing can be incorporated. For example, in one study, bootstrapping was used to evaluate whether the observed R_partial_^2^ for a given covariate was greater than would be expected by chance [91].

This method has been used to attribute portions of variability in metabolomic profiles to explanatory variables like study subject demographics and sample processing factors [91,92,93]. It has been applied frequently in studies of the European Prospective Investigation into Cancer and nutrition (EPIC) cohorts, where country and batch or sub-study membership have emerged as major contributors to variability, and have informed the interpretation of observed metabolomic differences [90,94,95,96]. The utility of this approach for identifying sources of variation has also led to its use in data processing pipelines, wherein the information gained by PC-PR2 analysis is used to guide normalization and enable pooling of metabolomics data across studies [97,98].

#### 3.2.4. Analysis of Variance–Simultaneous Component Analysis

Analysis of Variance–Simultaneous Component Analysis (ASCA) was developed specifically for the analysis of complex multivariate datasets [99,100,101]. This method extends the analysis of variance (ANOVA) framework to multivariate data by decomposing the original data matrix into a series of submatrices corresponding to the factors and interactions defined by the experimental design, along with a residual matrix. PCA is then performed on each submatrix to generate models that describe the overall effect of each factor or interaction. In this context, the PCA step is referred to as simultaneous component analysis (SCA) because component analysis is applied to the submatrices rather than the original, undecomposed data matrix. This decomposition also partitions the sums of squares in the original data matrix into factor-specific sums of squares. Under a balanced experimental design, these factor-specific sums of squares can be used to quantify the proportion of total variation explained by each factor and interaction [100]. These relative contributions can be compared across factors to determine which experimental variables account for the greatest proportions of variability in the dataset, as conceptually illustrated in Figure 7. In unbalanced designs, however, these values become less informative and may be biased, requiring modified ASCA approaches that are better suited for unbalanced data [102].

Like PCA, the scores and loadings of the resulting SCA models can be examined to extract information specific to the contribution of a given factor or interaction. The corresponding loadings indicate which metabolites contribute most strongly to the variation associated with each factor or interaction. The advantage of this approach over traditional PCA is that, because it is applied to decomposed data matrices, it can reveal smaller and subtler sources of variation that may not be observed in standard PCA. In addition, bootstrapping or permutation testing can be performed to assess the statistical significance of the observed factor and interaction contributions.

The value of ASCA has been demonstrated in untargeted metabolomics studies involving complex multifactorial and longitudinal experimental designs. For example, ASCA has been applied to investigate metabolic changes associated with neoadjuvant chemotherapy in breast cancer, where it enabled treatment-related metabolic variation to be separated from temporal and patient-specific sources of variability [103]. By decomposing the dataset according to experimental factors and interactions, ASCA can reveal subtle sources of variation that may not be observed using conventional PCA alone. As a result, ASCA is particularly well suited for high-dimensional omics datasets involving multiple interacting experimental variables, repeated measurements, or time-course analyses [104,105].

### 3.3. Comparison of Methods for Assessing Variability

The methods described above differ in the type of information they provide about variability within MS-based omics datasets. Some approaches identify general patterns of variability, while others assess the reproducibility of individual measurements or quantify the contribution of known experimental and biological factors. These differences are summarized in Table 1 and should be considered when determining which approaches are most appropriate for a given study.

When selecting a method, the first consideration should be the type of variability being investigated. If the source is unknown, exploratory approaches such as PCA, HCA, or distribution-based visualizations may provide an appropriate starting point. If the goal is to assess analytical reproducibility, CV, D-Ratio, or ICC may provide more useful quantitative information. Alternatively, when experimental or biological factors of interest are already known, gPCA, PVCA, PC-PR2, or ASCA can be used to determine whether these factors contribute to the observed variability and, depending on the method, quantify the magnitude of their contribution. ASCA is particularly suited for studies with a defined experimental design, as variability is partitioned according to the experimental factors included in the model.

Ultimately, there is no single method or quantitative threshold that can be used to assess variability across all MS-based omics studies. While established guidelines are available for some measures, such as CV and ICC, other measures must be interpreted in the context of the experimental design and the source of variability being evaluated. The use of complementary approaches can therefore provide a more comprehensive assessment of the variability present within a dataset.

## 4. Conclusions

Variability is an inherent and multifaceted feature of MS-based omics datasets. A comprehensive understanding of this variability is important for distinguishing meaningful biological differences from unwanted technical or experimental variation. The methods discussed in this review provide different approaches for detecting and quantifying these sources, which can ultimately provide a more complete understanding of the factors influencing an MS-based omics dataset.

Once a source of variability has been identified, the appropriate response will depend on its origin. Technical variability associated with sample preparation, batch effects, or instrument performance may indicate a need for greater standardization, normalization, or additional quality control measures. In contrast, biological variability may represent meaningful differences among samples and may instead require increased biological replication or consideration of relevant biological variables during statistical analysis. Therefore, simply detecting the presence of variability is not sufficient. Identifying its source and understanding its contribution to the dataset are important for determining whether the observed variation should be retained, accounted for, or minimized and for guiding appropriate decisions during experimental design and data processing.

As MS-based omics datasets continue to grow in size and complexity, approaches for assessing variability will need to evolve alongside them. One area of continued development is the automation of quality control, where multiple measures of data quality could be incorporated into data processing workflows to more efficiently identify problematic samples, features, or analytical runs. Machine learning may also provide opportunities for identifying complex patterns of variability that may not be readily detected using conventional approaches. These challenges may become particularly important as metabolomics is increasingly integrated with other omics platforms, where technical variability introduced by individual platforms must be distinguished from biological variation shared across multiple data types. Continued development of approaches for detecting, quantifying, and interpreting these different sources of variability will be important for ensuring that increasingly complex MS-based omics datasets can be appropriately evaluated and used to draw reliable biological conclusions.

## Figures and Tables

**Figure 1:**
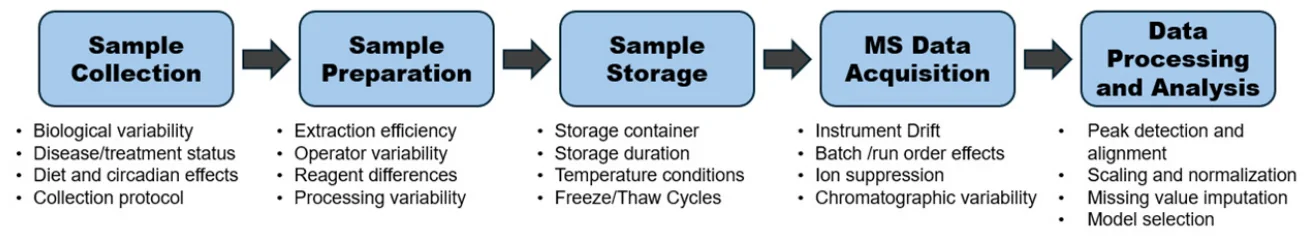
Sources of systematic variability in MS-based omics experiments. Schematic overview of example sources of variability that may be introduced across the experimental workflow, from sample collection to data analysis.

**Figure 2:**
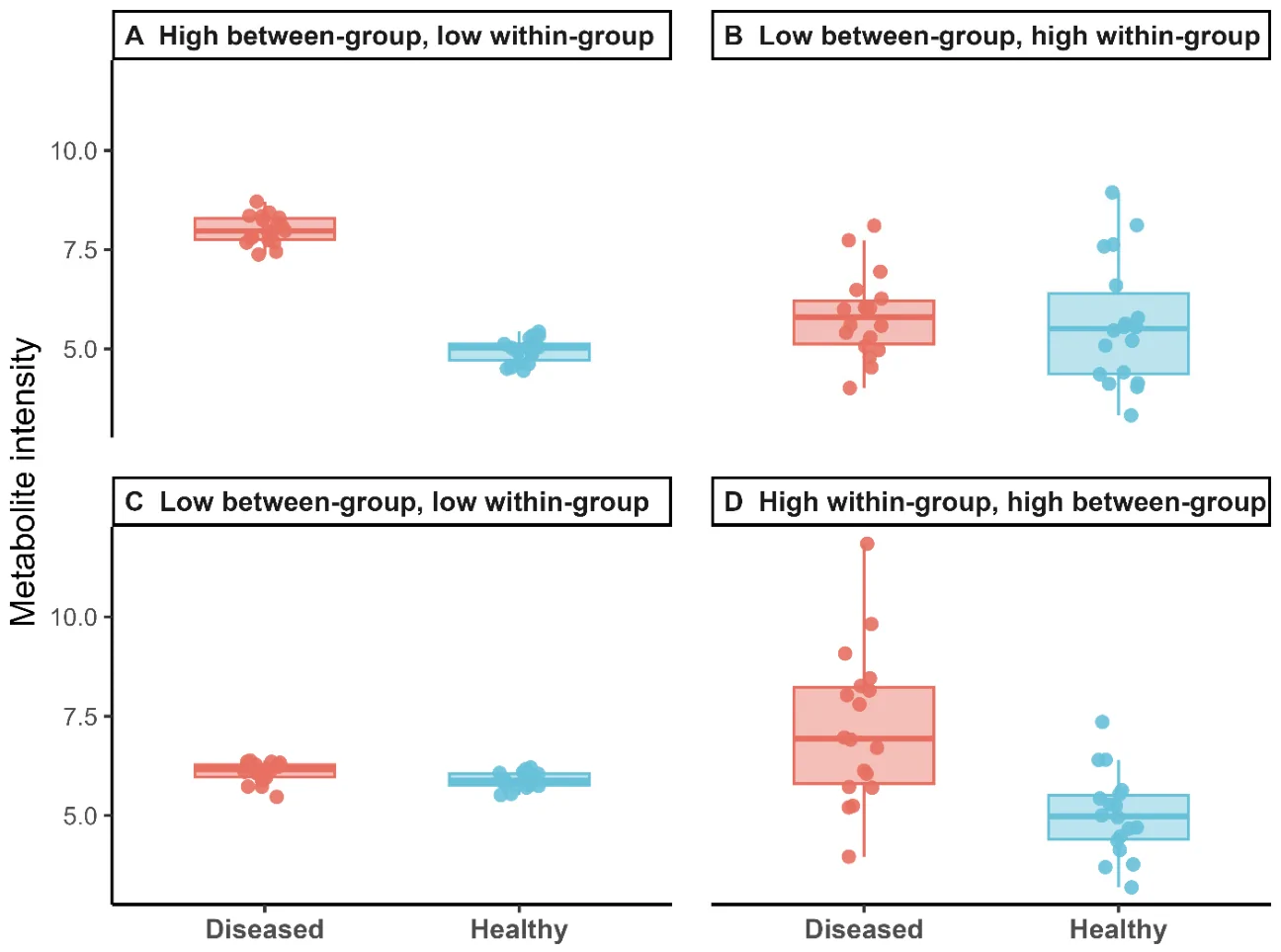
Effect of between-group and within-group variability. Boxplot examples illustrating how variation within and between groups affects the observed separation of healthy and diseased samples. (**A**) High between-group and low-within group variability results in clear separation between the two groups. (**B**) Low between-group and high within-group variability results in substantial overlap between groups. (**C**) Low between-group and low within-group variability produces tightly clustered groups with only a small difference between their distributions. (**D**) High within-group and high between-group variability produces greater dispersion within each group, which can make the difference between groups more difficult to distinguish.

**Figure 3:**
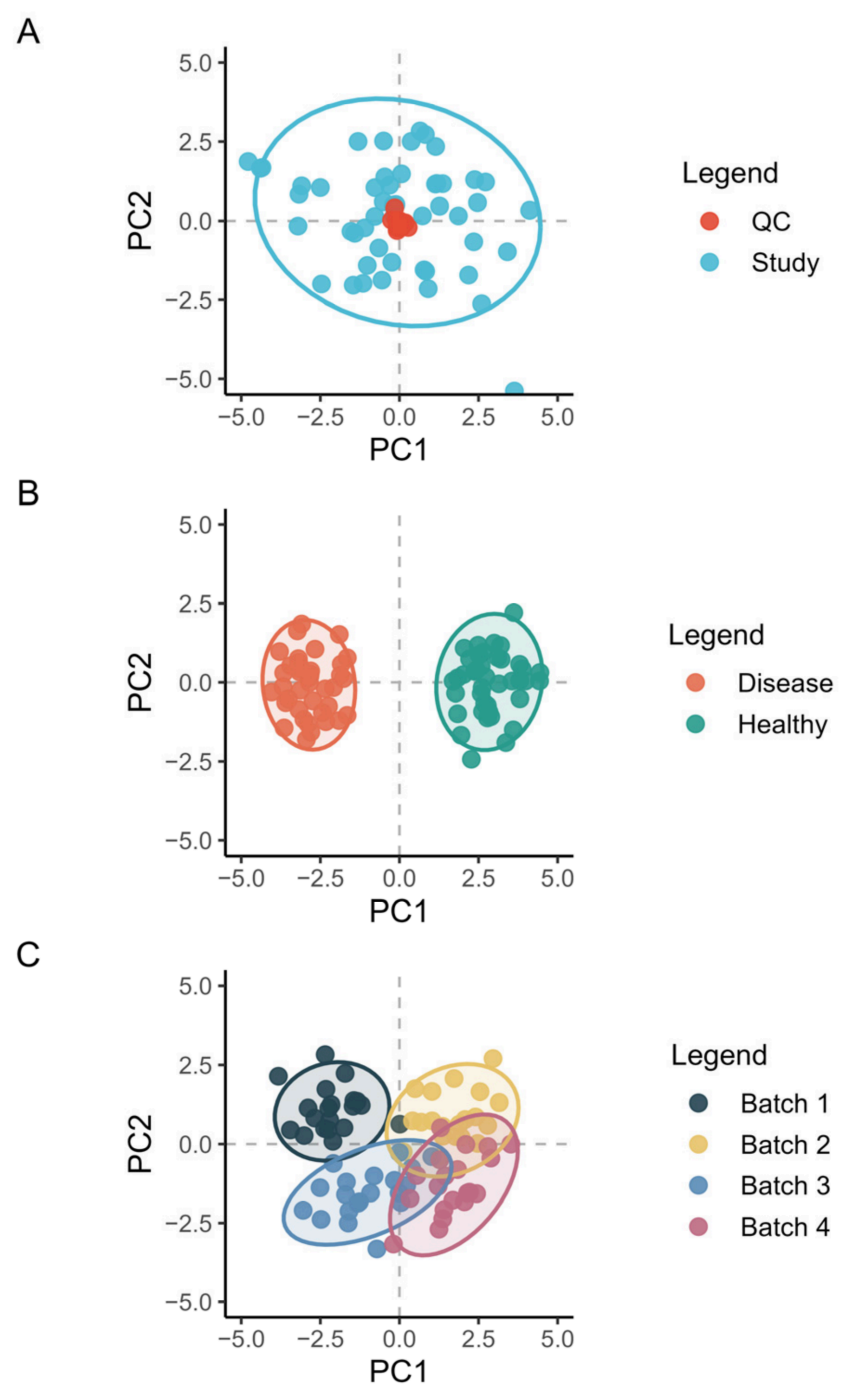
Principal component analysis for detecting sources of variability in MS-based omics studies. PCA score plots illustrating (**A**) good analytical reproducibility through tight clustering of QC samples relative to study samples, and study sample clustering driven by (**B**) biological variation or (**C**) batch-related variation.

**Figure 4:**
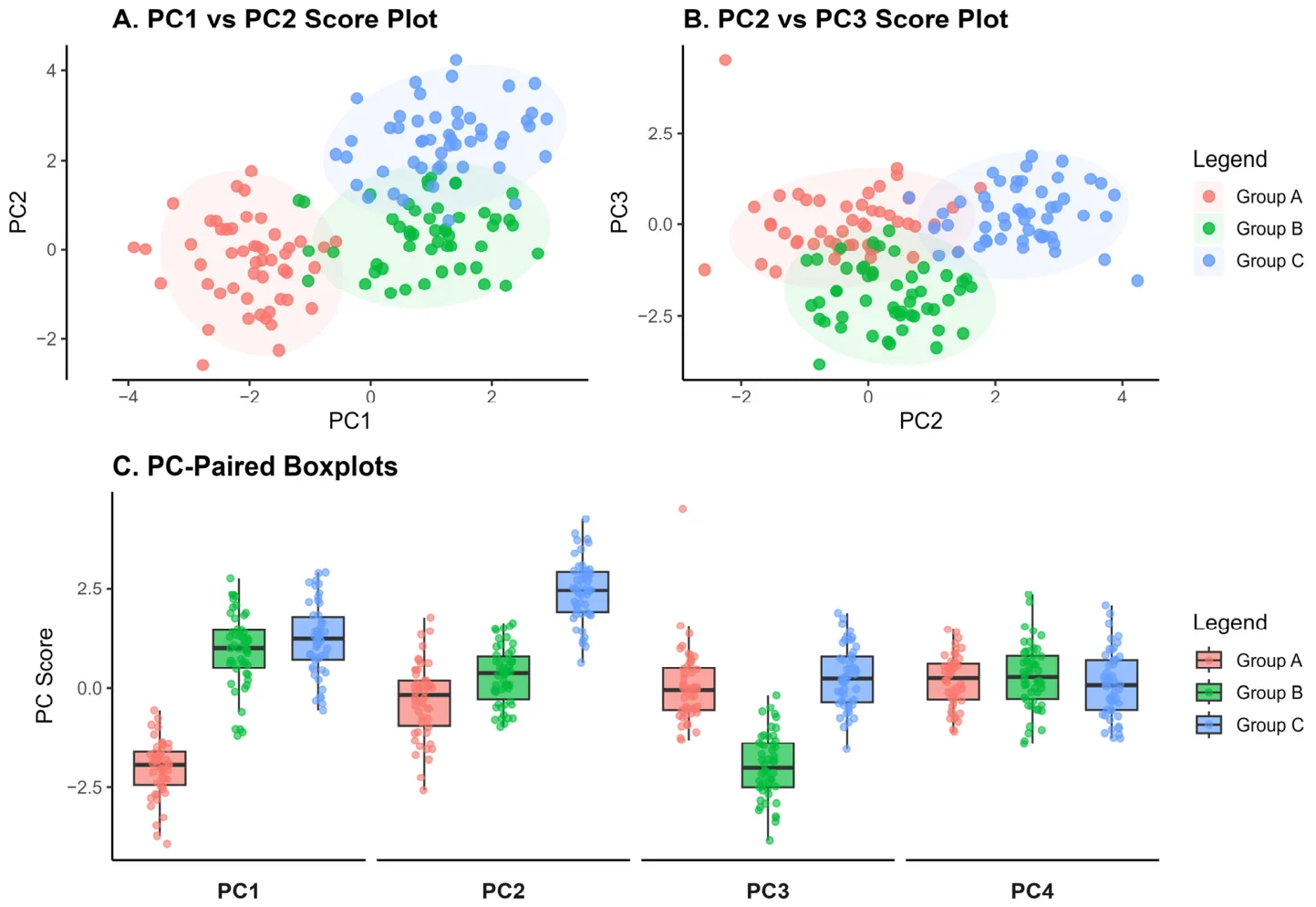
Different principal components can capture distinct patterns of variability in PCA. PCA performed on a simulated dataset containing three sample groups (Groups A–C) illustrates how patterns of variation may be distributed across multiple principal components. (**A**) PCA score plot of PC1 versus PC2, showing separation among the three sample groups based on the first two principal components. (**B**) Score plot of PC2 versus PC3, demonstrating that examination of an additional principal component can reveal different patterns of group separation that may not be apparent from PC1 and PC2 alone. (**C**) Boxplots of PC scores for Groups A–C across PC1-PC4, illustrating how the contribution of group-associated variability differs across individual principal components. These plots (made with hypothetical data for illustrative purposes) demonstrate that restricting interpretation to the first two principal components may overlook patterns of variability captured by higher-order components.

**Figure 5:**
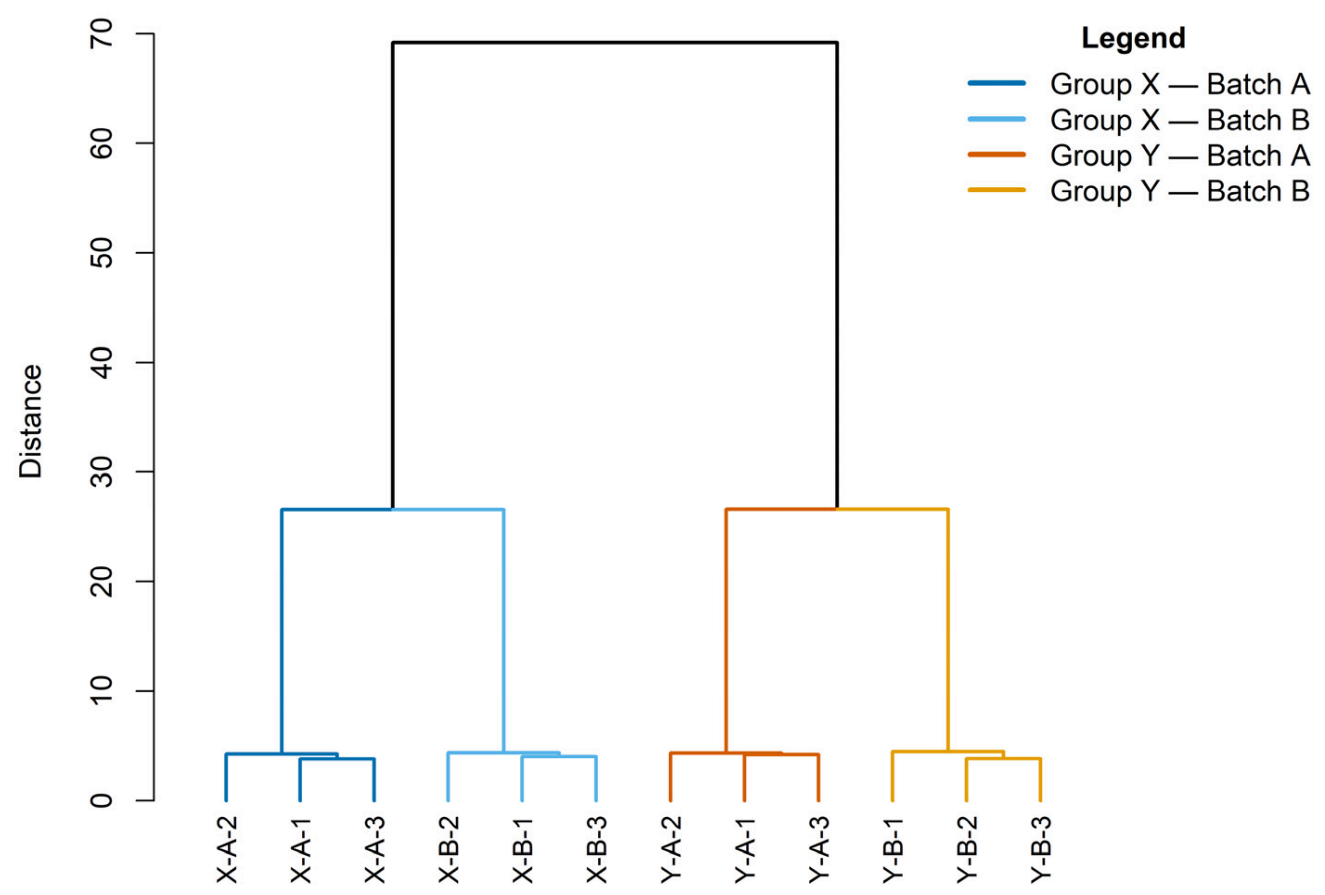
Hierarchical clustering reveals patterns of similarity among samples. Hierarchical cluster analysis (HCA) of simulated data containing triplicate samples from two sample groups (Groups X and Y) and two analytical batches (Batches A and B). Samples are grouped according to their similarity, with shorter distances indicating greater similarity between samples. In this example, samples primarily cluster according to sample group, while samples within each group further cluster according to analytical batch.

**Figure 6:**
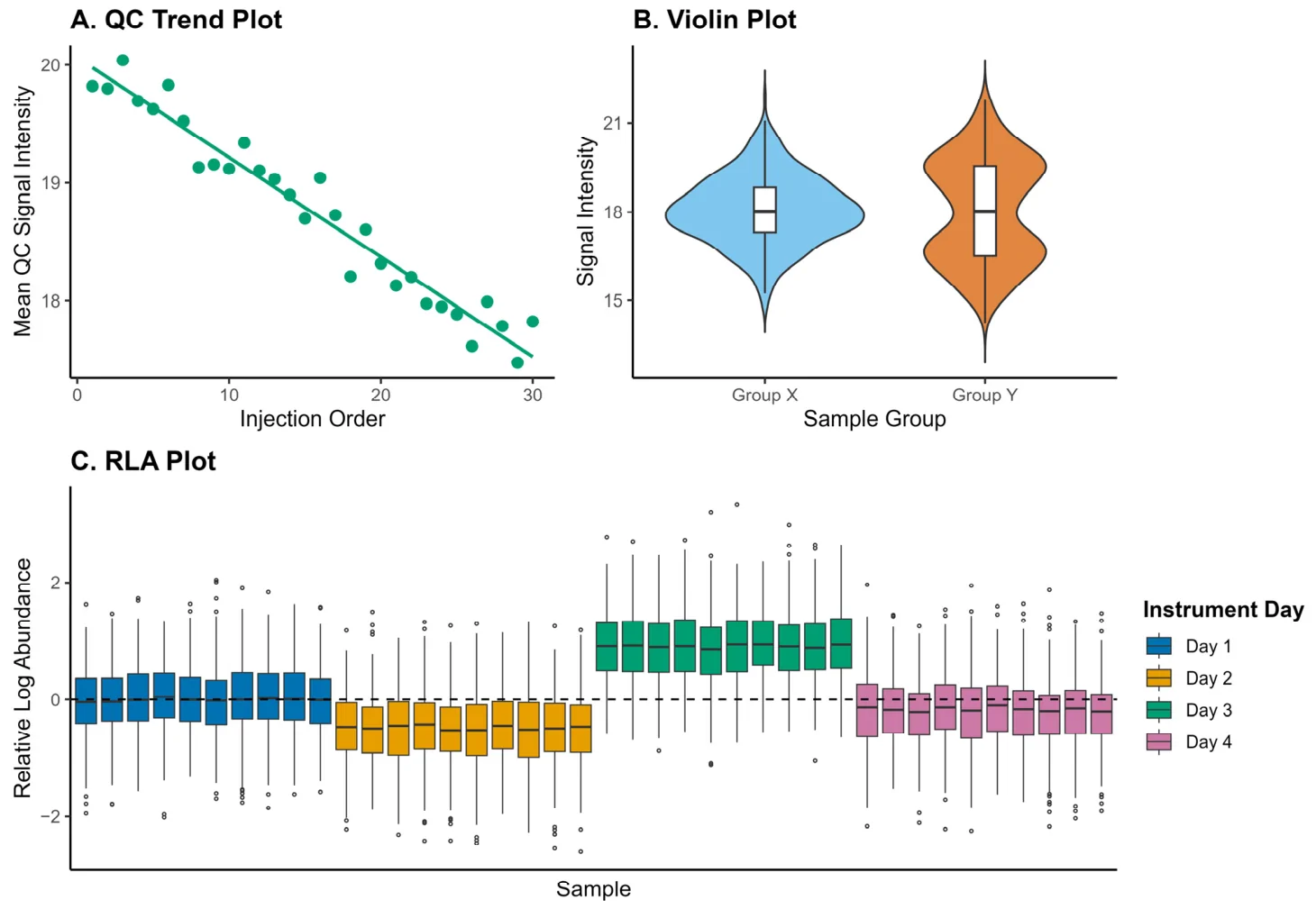
Distribution and trend plots for identifying variability in MS-based metabolomics data. (**A**) QC trend plot showing mean QC signal intensity as a function of injection order, where the decreasing signal illustrates potential instrumental drift over the course of an analytical run. (**B**) Violin plots showing the distribution of signal intensities for two sample groups, illustrating differences in the shape and spread of their intensity distributions. (**C**) Relative log abundance (RLA) plots showing feature-level abundance distributions across samples collected over four instrument days. Shifts in the RLA distributions between days illustrate how systematic analytical effects can be identified across groups of samples.

**Figure 7:**
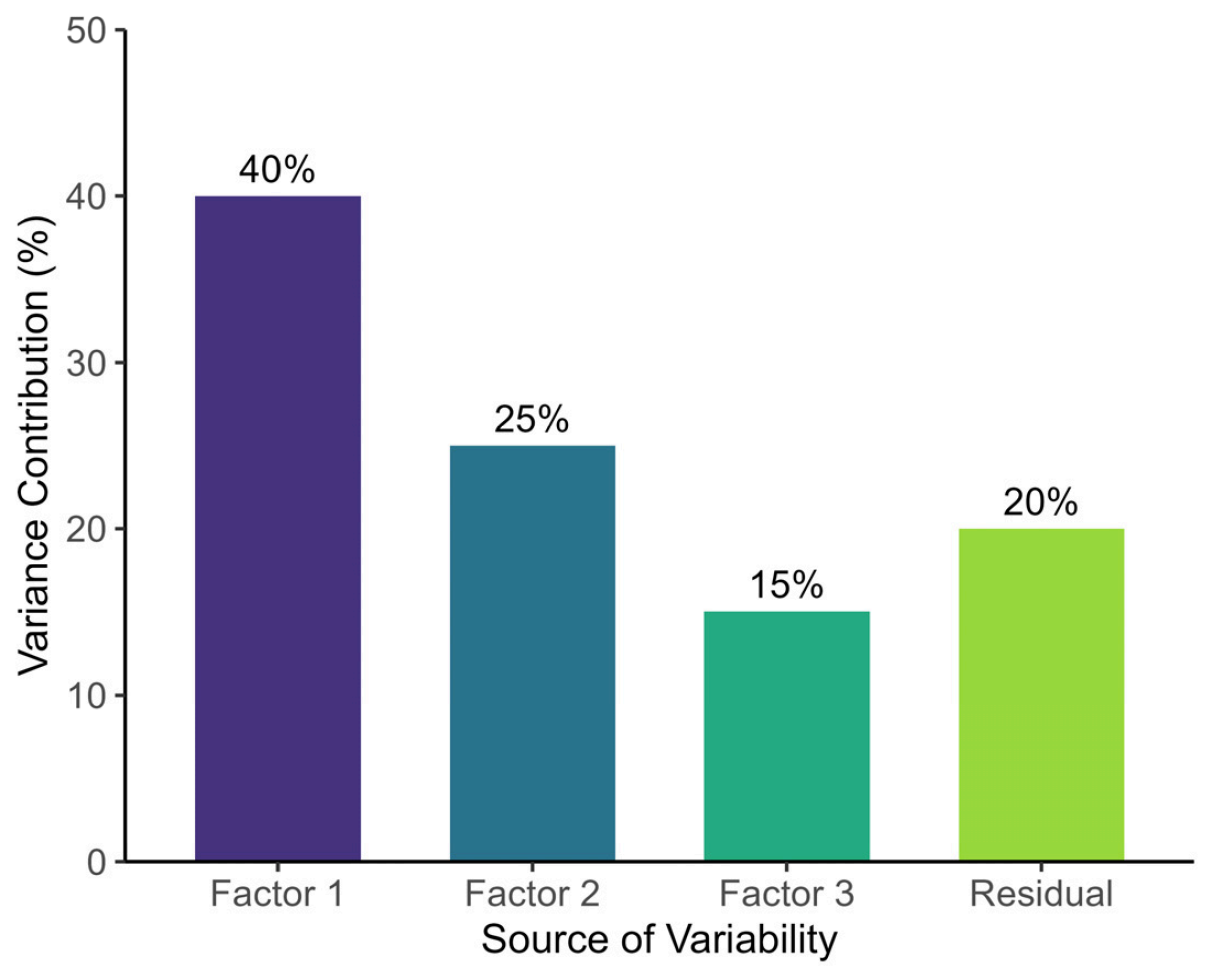
Variance Contributions of Different Sources of Variability. Conceptual illustration of the relative variance contributions associated with multiple factors. Factors represent potential experimental, technical, or biological sources of variability included in the analysis. Values are hypothetical and are provided for illustrative purposes.

**Table 1:** Comparison of methods for assessing variability in MS-based omics datasets.

Method	What It Assesses	Interpretation	References
PCA	Identify major patterns of variability and sample clustering	Clustering or separation can indicate systematic patterns of variability and, when interpreted alongside metadata, may suggest potential sources of variation	[24,27]
CV/RSD	Assess variability and analytical reproducibility of individual features	Higher values indicate greater relative variability	[30]
HCA	Identify similarities and groupings among samples	Clustering can reveal samples with similar patterns of variation	[59,60,61]
Distribution/Trend Plots	Identify systematic trends or changes across samples	Shifts or trends can indicate systematic variability	[67,68,69]
D-Ratio	Compare technical variability with overall study variability	Lower values indicate that technical variability is small relative to overall variability	[30,51]
ICC	Assesses measurement reliability by comparing biological and technical sources of variability	Higher values indicate that biological differences are large relative to technical variability	[7,74]
gPCA	Evaluate variability associated with a known factor	Higher δ values indicate a stronger contribution of the factor to overall variability; statistical significance can be assessed by permutation testing	[79]
PVCA	Quantify contributions from multiple sources	Larger percentages indicate that a factor accounts for a greater proportion of total variability	[83,84]
PC-PR2	Quantify variability associated with multiple factors	Larger variance contributions indicate a greater influence of the corresponding factor	[90]
ASCA	Quantify and visualize variability associated with factors defined in the experimental design	Variance contribution describes effect magnitude; permutation testing can determine statistical significance	[99,101]

## Data Availability

No new data were created or analyzed in this study. Data sharing is not applicable to this article.
